# Isolation and Characterization of the Wastewater Micropollutant Phenacetin-Degrading Bacterium *Rhodococcus* sp. Strain PNT-23

**DOI:** 10.3390/microorganisms11081962

**Published:** 2023-07-31

**Authors:** Yaxuan Yuan, Kexin Wang, Yihe Liu, Maoting Jiang, Yinhu Jiang, Jiguo Qiu

**Affiliations:** Key Laboratory of Agricultural Environmental Microbiology, Ministry of Agriculture, College of Life Sciences, Nanjing Agricultural University, Nanjing 210095, China

**Keywords:** phenacetin, micropollutant, biodegradation, *Rhodococcus*, catabolic intermediates

## Abstract

Phenacetin, an antipyretic and analgesic drug, poses a serious health risk to both humans and aquatic organisms, which is of concern since this micropollutant is frequently detected in various aquatic environments. However, rare pure bacterial cultures have been reported to degrade phenacetin. Therefore, in this study, the novel phenacetin-degrading strain PNT-23 was isolated from municipal wastewater and identified as a *Rhodococcus* sp. based on its morphology and 16S rRNA gene sequencing. The isolated strain could completely degrade 100 mg/L phenacetin at an inoculum concentration of OD_600_ 1.5 within 80 h, utilizing the micropollutant as its sole carbon source for growth. Strain PNT-23 exhibited optimal growth in LB medium at 37 °C and a pH of 7.0 with 1% NaCl, while the optimal degradation conditions in minimal medium were 30 °C and a pH of 7.0 with 1% NaCl. Two key intermediates were identified during phenacetin biodegradation by the strain PNT-23: N-acetyl-4-aminophenol and 4-aminophenol. This study provides novel insights into the biodegradation of phenacetin using a pure bacterium culture, expands the known substrate spectra of *Rhodococcus* strains and presents a potential new candidate for the microbial removal of phenacetin in a diverse range of environments.

## 1. Introduction

Phenacetin is an acetamide antipyretic and analgesic drug, which has been used historically in both human and veterinary medicine. Introduced in 1897, phenacetin is considered the first synthetic pharmaceutical drug [1,2,3]. However, phenacetin and its derivatives pose potential threats to human and environmental health. Studies have found that the use of phenacetin is associated with cardiotoxicity and retinopathy in patients [3,4]. Additionally, the oxidation product of phenacetin, 5-methoxy-phenoxetine, has been found to cause liver damage and potentially induce cancer [5]. Phenacetin was listed as a class I carcinogen by the International Agency for Research on Cancer (IARC) of the World Health Organization (WHO) in 2012 [6]. Although the WHO officially banned the use of phenacetin in 2018, phenacetin-containing drugs have not been banned altogether and can still be used in compound medicines with other components (such as aminopyrine), resulting in their continued use with a phase-out approach in some regions, such as mainland China and Canada. Furthermore, phenacetin is still employed as a targeted drug in pharmaceutical research [7]. Due to its low cost and high availability, phenacetin is often used as an adulterant for cocaine [8,9], increasing the risk of kidney damage and cancer for cocaine abusers. Phenacetin is a recalcitrant compound that cannot be completely decomposed by the human body, with phenacetin residues continuing to be detected in sewage wastewater [10] even after its ban. Phenacetin has been frequently detected as an aquatic micropollutant at concentrations in the ng/L to μg/L range in various regions worldwide [11], including Japanese drinking water purification plants (44 ng/L) [12] and Chinese wastewater treatment plants (WWTPs) (8.22 μg/L) [13]. In South Africa’s Kwa-Zulu Natal province, surface water samples were found to contain phenacetin concentrations ranging from 1.95 to 68.3 μg/L [14]. Phenacetin residues in the environment are known to be toxic to *Chlorella*, *Selenastrum*, nitrite-oxidizing bacteria (*Nitrospira* spp.) and other aquatic organisms and to inhibit their growth [6,15,16]. In addition, the downstream metabolite of phenacetin, acetaminophen, is also harmful to aquatic ecosystems. Studies have shown that acetaminophen can cause genotoxicity in fish and other aquatic organisms, as well as kidney damage. Therefore, phenacetin not only poses a direct risk to human health, but it also has the potential to cause harm to organisms throughout the food chain [17]. Therefore, considering the high risk to human health and non-target organisms, it is necessary to implement effective methods for the removal of phenacetin from a diverse range of environments.

Various methods have been studied for the degradation of phenacetin, including UV oxidation [18], advanced oxidation processes (AOPs) [19] and chemical oxidation [20]. For example, UV–chlorine systems have been proven to be effective at breaking down phenacetin into non-toxic byproducts, with the addition of UV irradiation and chlorine ions significantly impairing the degradation efficiency [21]. AOPs can generate hydroxyl radicals with strong oxidation capabilities and the capacity to degrade phenacetin [21]. In terms of chemical oxidation approaches, various systems have been shown to effectively degrade phenacetin in water, such as acid-washed zero-valent aluminum, different free radicals (HO^−^, SO_4_^−^ and CO_3_^−^) [6] and a CuFe_2_O_4_ magnetic catalyst combined with ozone [5]. However, these methods have some notable limitations, with UV oxidation and AOPs requiring high energy inputs and expensive equipment, while oxidant treatments require high chemical oxidant concentrations, which may result in secondary pollution. In comparison, biodegradation offers a more environmentally friendly and economically feasible option for the removal of phenacetin from various environments. Biodegradation utilizes microorganisms with the capacity to metabolize phenacetin and convert it into harmless substances, making it a promising approach for managing phenacetin pollution in a safe and sustainable manner. Phenacetin has been shown to be relatively easily biodegradable compared to many other pharmaceutical pollutants [22,23]. For example, Li et al. [22] found that phenacetin could be degraded effectively using an A2/O (anaerobic–anoxic–aerobic) bioreactor, while Longli Bo et al. [11] demonstrated that up to 85% of phenacetin could be removed by biological WWTP processes (Xi’an City, China). The biodegradation of phenacetin is a sustainable solution that minimizes the impact of phenacetin on the environment [23]. Hart et al. found that *Penicillium* species are capable of degrading phenacetin and its derivatives [24,25]. However, there have been no reports to date of pure bacterial strains capable of degrading phenacetin. Furthermore, the bacterial pathways and mechanisms used for phenacetin degradation remain unknown.

By investigating and characterizing bacterial isolates capable of phenacetin biodegradation, insights can be gained into the mechanisms and pathways involved in the phenacetin degradation process. This knowledge is critical for developing and optimizing bioremediation strategies for phenacetin-contaminated environments.

In this study, a newly isolated bacterium capable of degrading phenacetin, *Rhodococcus* sp. strain PNT-23, was isolated from municipal wastewater samples. The catabolic processes involved in the utilization of phenacetin by strain PNT-23 were characterized, and key intermediate products were identified during the degradation process. The findings of this study provide scientific evidence and technical support for the remediation of phenacetin pollutants using pure culture, promoting the development of efficient and sustainable approaches for environmental micropollutant bioremediation.

## 2. Materials and Methods

### 2.1. Chemicals and Media

Phenacetin (99% purity) and PCR primers were purchased from Macklin Biochemical (Shanghai, China). Prior to use, phenacetin was dissolved in deionized water and filter sterilized. Methanol was used for high-performance liquid chromatography (HPLC) analysis (Honeywell International Co., Charlotte, NC, USA). Luria–Bertani (LB) medium, containing tryptone (10.0 g/L), yeast extract (5.0 g/L) and NaCl (10.0 g/L) at a pH of 7.0, was used to enrich bacteria. Mineral salt medium (MSM) was used for bacterial growth and isolation, containing (NH_4_)_2_SO_4_ (1.0 g/L), KH_2_PO_4_·2H_2_O (0.5 g/L), MgSO_4_·7H_2_O (0.2 g/L), K_2_HPO_4_·3H_2_O (1.5 g/L) and NaCl (1.0 g/L) at a pH of 7.0. To prepare the corresponding solid media, 1.8% agar powder was added to the liquid media. All culture media were sterilized using an autoclave at 121 °C for 25 min.

### 2.2. Bacterial Enrichment and Isolation

Municipal wastewater samples were collected from a site located in Nanjing City, China. For bacterial enrichment, 250 mL of MSM was prepared and supplemented with 100 mg/L phenacetin as the sole source of carbon and energy. Then, 10 mL of the wastewater sample was used to inoculate the MSM, which was incubated at 30 °C with continual shaking at 180 rpm for 7 days. The phenacetin concentration in the enrichment culture was determined by UV/Vis spectrophotometry. The enrichment process was repeated five times to ensure the enrichment of cultures with phenacetin-degrading strains [26,27], after which the enriched culture was serially diluted and spread onto MSM agar plates containing 100 mg/L phenacetin and incubated at 30 °C for 3–7 days until colonies appeared. Different colonies were selected from each MSM plate and isolated by streak plating, with this process repeated three consecutive times to ensure that the selected colonies were pure and not contaminated [28]. After three rounds of streak plating, single colonies were isolated and cultivated, and their phenacetin degradation capabilities were verified.

Whole-cell catalysis experiments were performed using intact bacterial cells to verify their biocatalytic phenacetin degradation performance [29,30]. The selected strain was grown in LB medium until the cultures’ optical absorbance at 600 nm (OD_600_) reached 1.5, at which point 1 mL of bacterial solution was added to MSM containing 100 mg/L phenacetin. The mixture was then centrifuged at 5000× *g* for 5 min to remove any cell debris or other insoluble matter. The deposit was resuspended in 2 mL ddH_2_O, and the previous steps were repeated. Afterward, the mixture was centrifuged again at the same speed and duration and then resuspended in 2 mL of MSM. The treatment groups consisted of bacterium, MSM and phenacetin, while the control group consisted of MSM and phenacetin only. All samples were incubated at 30 °C with continual shaking at 180 rpm. Bacterial suspension samples (700 μL) were collected at different time points for analysis, and they were centrifuged at 13,000× *g* for 3 min. The supernatant was collected, and 300 μL was mixed with 600 μL of UV dilution solution (0.1 M HCl) as required for UV/Vis determination. Since phenacetin has a characteristic absorption peak at 244 nm, the samples were analyzed using a UV/Vis spectrophotometer (UV-2450, Shimadzu, Japan) at a wavelength of 200–350 nm [31]. Ultimately, a high-efficiency phenacetin-degrading strain was obtained and named PNT-23.

### 2.3. Identification of Strain PNT-23

To identify the PNT-23 strain, various methods were used to observe its physicochemical properties. Gram staining was employed to determine whether the bacterium was Gram positive or negative. The growth characteristics of the strain were described according to colony morphology and adhesion characteristics on different media. Antibiotic susceptibility testing was performed to establish the resistance profile of the strain, using eight antibiotics: tetracycline, gentamicin, kanamycin, chloramphenicol, streptomycin, ampicillin, lincomycin and spectinomycin. To evaluate the carbon source utilization profile of the strain, it was inoculated into various types of liquid media containing different carbon sources (sugars, glucose, citrate and acetate), and the growth of the strain was measured over time. Finally, genomic DNA was extracted from strain PNT-23 using a bacterial genomic DNA mini-kit from Vazyme (Nanjing, China). Universal primers 27F (5′-AGAGTTTGATCMTGGCTCAG-3′) and 1492R (5′-TACGGYTACCTTGTTACGACTT-3′) that target conserved regions of the bacterial genome were used to amplify 16S rRNA genes [32]. The PCR reaction conditions were set as follows: initial denaturation at 95 °C for 5 min, followed by 30 cycles of denaturation at 94 °C for 1 min, annealing at 52 °C for 1 min, extension at 72 °C for 2 min and a final extension at 72 °C for 10 min. High-fidelity DNA polymerases were added to ensure accurate amplification of target sequences. The PCR products were sequenced by Shanghai Sangon Biotech Co., Ltd. (Shanghai, China), and the resulting sequence was identified by BLAST comparison. To confirm the correct identification of strain PNT-23 and determine its phylogenetic relationship with other closely related strains, a neighbor-joining phylogenetic tree was constructed using MEGA software, version 11.0. Reference 16S rRNA gene sequences of aligning model strains were obtained from GenBank (https://www.ncbi.nlm.nih.gov/ accessed on 23 April 2023) and EzTaxonserver (http://www.eztaxon.org accessed on 23 April 2023). To ensure reliability, a strain from a distinct genus was considered an outgroup. The 16S rRNA gene sequences were aligned using the ClustalW algorithm, and a phylogenetic tree was constructed based on the maximum composite likelihood model. The reliability of the tree was assessed using bootstrap analysis with 1000 replicates.

### 2.4. Characteristics of Phenacetin Biodegradation by Strain PNT-23

To investigate the characteristics of phenacetin biodegradation by strain PNT-23 in detail, its growth was evaluated under various conditions. Specifically, PNT-23 biomass was measured in LB medium by determining its OD_600_ as a reflection of the cell growth status of the strain, with varying temperatures of 16, 20, 25, 30, 37 and 44 °C; pH values of 5.0, 6.0, 7.0, 8.0, 9.0 and 10.0; and NaCl concentrations from 1% to 10% (*w*/*v*). The parameter variation ranges were selected based on the typical conditions required by bacteria, and determining the optimal temperature, pH and salt concentration lays a foundation for the successful culture of drug-degrading strains. Sampling was performed every 12 h, observing the growth status within an overall 96-h period, thus determining the optimal growth conditions for PNT-23. Subsequently, the cells were washed twice with MSM and suspended at an OD_600_ of 2.0. Fresh bacterial solutions (1% inoculum) were then added to 100 mL of MSM containing 100 mg/L phenacetin in 250-mL Erlenmeyer flasks. The bacterial cell density was determined in MSM during the degradation process using the dilution spread plate method, calculating the number of colony-forming units per milliliter (CFU/mL) [33]. The effect of temperature on phenacetin degradation was studied by conducting growth tests at five temperature levels (15, 20, 30, 37 and 45 °C) while maintaining a pH of 7.0. The culture medium was sampled at 8-h intervals. Subsequently, to explore the influence of pH on the degradation process, cultures were maintained at the identified optimal temperature, and the pH value was adjusted to 5.0, 6.0, 7.0, 8.0, 9.0 and 10.0 using HCl and NaOH. Periodic sampling was carried out over the 96-h period at 12-h intervals to determine the effect of different pH conditions on phenacetin degradation and to identify the optimal pH for phenacetin degradation by PNT-23. Additionally, the optimal salt ion concentration for phenacetin degradation by strain PNT-23 was determined by evaluating the effects of different NaCl concentrations (1%, 2.5%, 5.0%, 7.5% and 10% [*w*/*v*]) at the previously established optimal temperature and pH. All samples were tested in triplicate to ensure the accuracy and reproducibility of the results. The determination of optimal conditions provided a sufficient basis to study the metabolites and pathways of phenacetin.

### 2.5. Detection of Phenacetin Metabolites and Analysis Methods

UV/Vis spectrophotometry was used to preliminarily identify the metabolic intermediates formed during phenacetin degradation, with HPLC and LC-TOF/MS analysis performed to identify metabolic intermediates by comparison against commercially available standards and a mass spectra library, respectively. Samples were collected from the culture flasks at regular 4-h intervals over a 48-h period. For HPLC analysis, all samples were collected via centrifugation at 13,000× *g* for 5 min, followed by filtration of the upper layer of solution using a 0.22-μm injection filter. An Agilent TC-C18 column was used (4.6 mm × 250 mm (i.d.) and a particle size of 5 μm), with the mobile phase consisting of methanol and ultrapure water (ratio of 15%:85% (*v*:*v*)) with 1% formic acid at a flow rate of 0.8 mL/min. The column temperature was maintained at 30 °C, and the injection volume was set at 20 μL, with absorption peaks monitored by diode array detection. To identify the metabolic intermediates of phenacetin, the supernatant was lyophilized, dissolved using methanol and then transferred to a Thermo UltiMate 3000 titanium system, consisting of a C18 reverse-phase column (250 × 4.60 mm, 5 μm) (Thermo Fisher Scientific, Waltham, MA, USA) and a liquid chromatography time-of-flight mass spectrometer (LC-TOF/MS) [31]. Positive ion patterns were analyzed to identify intermediates of phenacetin metabolism compared to standard samples, providing valuable insights into the phenacetin biodegradation process.

### 2.6. Calculations and Statistical Analysis

The extent of phenacetin degradation was determined using Equation (1) as follows:

Degradation extent (%) = (C_ck_ − C_t_)/C_ck_ × 100% (1)

where C_ck_ and C_t_ indicate the concentrations (mmol^−1^) of substrates in the control and treatment samples, respectively; and t (hour) indicates the incubation time.

## 3. Results and Discussion

### 3.1. Isolation and Identification of Phenacetin-Degrading Bacteria

**Figure 1:**
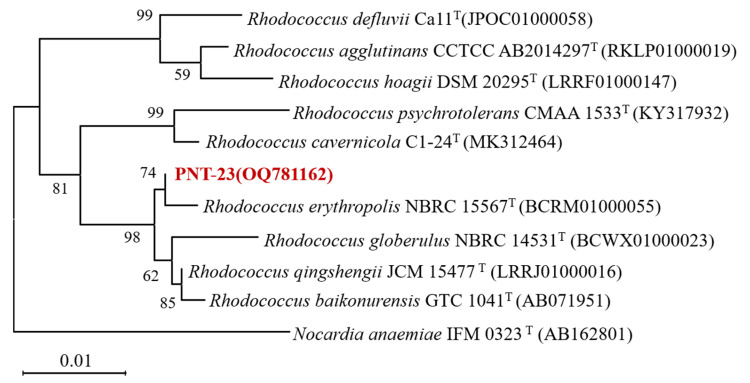
Phylogenetic tree based on the 16S rRNA gene sequences of strain PNT-23 and related taxa. The numbers displayed in the phylogenetic tree represent the bootstrap values, which were obtained from 1000 repeats.

Phenacetin was used as the sole carbon source for the enrichment culture, with the aim of isolating microbial strains capable of its degradation. Fifth-generation enrichment solutions were subjected to gradient dilution, with continuous streaking and purification using MSM plates containing 100 mg/L phenacetin as the sole carbon source, resulting in the isolation of a single strain named PNT-23. Strain PNT-23 is a Gram-positive bacterium that forms yellow, circular colonies with a slightly convex surface, exhibiting weak adhesion to medium and drying surfaces. Furthermore, strain PNT-23 displays resistance to most antibiotics and the capacity to utilize various carbon sources, including sugars, glucose, citrate and acetate. To confirm strain identification and determine the phylogenetic relationships between PNT-23 and other closely related strains, a neighbor-joining phylogenetic tree was constructed, including *Nocardia anaemiae* IFM as an outgroup to ensure the reliability of the analysis. The 16S rRNA gene sequencing results were screened against the BLAST database, indicating that strain PNT-23 was most closely related to species in the genus *Rhodococcus*, exhibiting the highest degree of similarity to *Rhodococcus baikonurensis* (99.76%) and *Rhodococcus erythropolis* (99.38%) (Figure 1). Based on the morphological characteristics and phenotype, combined with the results of 16S rRNA gene phylogenetic analysis, strain PNT-23 was identified as a *Rhodococcus* sp. The 16S rRNA gene nucleotide sequence of strain PNT-23 was submitted to GenBank under accession number OQ781162.

The environmental impact of pharmaceutical pollutants has become a growing global concern, with the ability of certain *Rhodococcus* bacteria to degrade these compounds becoming a major research focus [34,35,36]. Previous studies have reported the diverse catabolic pathways that enable *Rhodococcus* spp. to degrade various pharmaceutical compounds, such as paracetamol degradation by *Rhodococcus erythropolis* BIOMIG-P1 [37], ibuprofen degradation by *Rhodococcus cerastii* IEGM 127 [38] and acetylsalicylic acid degradation by *Rhodococcus jostii* IEGM 60 [39]. Additionally, *Rhodococcus* spp. display the ability to produce active enzymes, including esterases, lipases and hydroxylases, which could potentially enhance their capacity to degrade pollutants. Furthermore, the versatility of *Rhodococcus* spp. in terms of carbon source utilization makes them promising candidates for bioremediation purposes. While previous studies have explored the biodegradation of phenacetin, the existence of pure bacterial strains capable of degrading this drug has not been documented until now. This study provides the first report of a pure culture *Rhodococcus* strain capable of utilizing phenacetin as a sole carbon source. This finding expands the known spectrum of drugs that can be targeted by bioremediation using *Rhodococcus* spp., highlighting the potential key role of this genus in the bioremediation of environments contaminated with phenacetin and other micropollutants. Therefore, these findings demonstrate the importance of exploring the bioremediation potential of this versatile bacterial genus.

### 3.2. Growth and Phenacetin Degradation Characteristics of Strain PNT-23

#### 3.2.1. Growth Characteristics of Strain PNT-23

**Figure 2:**
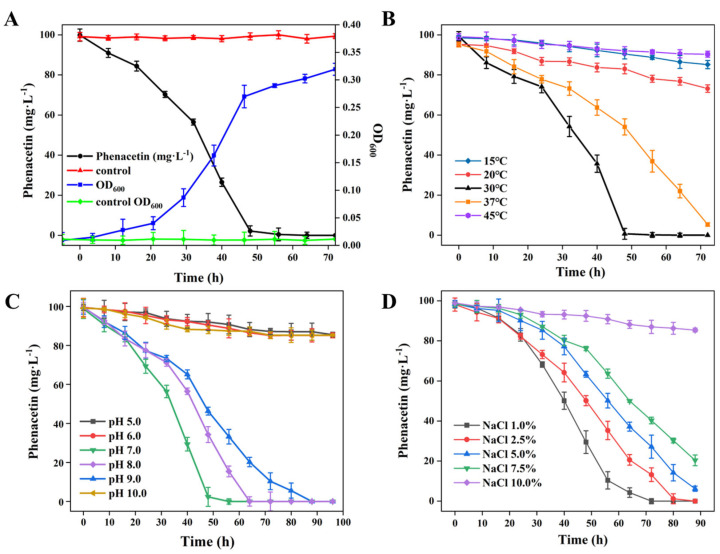
Phenacetin biodegradation performance of strain PNT-23 under varying conditions: (**A**) degradation of phenacetin by strain PNT-23 at a 1% inoculum level (OD_600_ = 1.5) in comparison to the control group consisting of only 100 mg/L phenacetin in MSM medium; (**B**) effects of different temperatures on phenacetin biodegradation by strain PNT-23; (**C**) effects of varying pH levels ranging from 5.0 to 10.0 on phenacetin biodegradation by strain PNT-23 under optimal temperature conditions; and (**D**) effects of NaCl concentrations ranging from 1% to 10% (*w*/*v*) on phenacetin biodegradation by strain PNT-23 under optimal temperature and pH conditions.

To evaluate the growth profile of strain PNT-23 and its ability to degrade phenacetin, tests were conducted using a 1% bacterial suspension inoculated into 100-mL sterile MSM with 100 mg/L phenacetin at a pH of 7.0. The flask was then cultured at 30 °C with continual shaking at 180 rpm. As shown in Figure 2A, strain PNT-23 was able to completely deplete 100 mg/L phenacetin within 72 h, corresponding to 100% removal. Simultaneously, the bacterial cells grew steadily, reaching a density of 2.57 × 10^8^ CFU/mL (Figure 2A). The growth rate of strain PNT-23 was slow during the first 2 h due to a period of adaptation to the medium. However, after 32 h, a significant increase in growth rate was observed, indicating that PNT-23 effectively utilized phenacetin as a carbon source for cell growth, with the highest phenacetin degradation rate occurring during the logarithmic growth phase. After 48 h, phenacetin was completely degraded, with the depletion of available carbon resulting in strain PNT-23 exhibiting a reduced growth rate. Thereafter, the development and reproduction of strain PNT-23 entered a stable stage. Overall, the optimal growth conditions for PNT-23 were determined to be 37 °C and a pH of 7.0 with a 1% salt concentration (Supplementary Figure S1).

#### 3.2.2. Effect of Temperature on Phenacetin Degradation

Temperature plays a crucial role in the degradation of phenacetin in natural environments. As illustrated in Figure 2B, complete removal of 100 mg/L phenacetin was achieved within 48 h by strain PNT-23 at 30 °C and within 72 h at 37 °C (with all other parameters remaining constant). Conversely, at 15 °C, 20 °C and 45 °C, the extents of phenacetin degradation were 25.3%, 13.8% and 5.4%, indicating that strain PNT-23 has a limited temperature adaptation range and corresponding biodegradation potential. The extent of phenacetin degradation was ranked in the following order: 30 °C > 37 °C > 20 °C > 15 °C > 45 °C (Figure 2B). Specifically, these results suggest that phenacetin degradation by the PNT-23 bacterial strain occurs in a temperature-dependent manner, whereby excessively high or low temperatures can negatively impact the extent of phenacetin degradation. The observed temperature dependency of the extent of phenacetin degradation could be attributed to the mesophilic nature of bacterial strain PNT-23 and the temperature sensitivity of the enzymes involved in the phenacetin biodegradation process. Therefore, attention should be paid to the temperature range of the remediation environment during treatment to ensure the high degradation capability of PNT-23.

#### 3.2.3. Effect of pH on Phenacetin Degradation

The effect of varying pH conditions (5.0, 6.0, 7.0, 8.0, 9.0 and 10.0) on the degradation of phenacetin by strain PNT-23 was investigated at 30 °C. As shown in Figure 2C, complete phenacetin removal was achieved by strain PNT-23 under initial pH levels of 7.0, 8.0 and 9.0, with the optimum pH for phenacetin degradation determined to be 7.0. The phenacetin degradation extents were ranked in the order of pH 7.0 > pH 8.0 > pH 9.0 (Figure 2C), suggesting that strain PNT-23 is suitable for phenacetin degradation in neutral to slightly alkaline environments. In contrast, under initial pH levels of 5.0 and 10.0, PNT-23 achieved phenacetin degradation extents of 13.4% and 13.6%, with this low degradation ability indicating the harmful effects of extremely acidic or alkaline conditions on the performance of strain PNT-23. Since the natural growth environment of the bacteria is primarily neutral, the experimental conditions were standardized at a pH of 7.0, while in practical applications, the pH can be properly adjusted to maintain a weakly alkaline environment and achieve maximum degradation efficiency.

#### 3.2.4. Salt Tolerance of Strain PNT-23

Figure 2D illustrates the biodegradation of phenacetin by strain PNT-23 in the presence of varying initial NaCl concentrations of 1.0%, 2.5%, 5.0%, 7.5% and 10%. The results showed that PNT-23 achieved good degradation performance in the NaCl range of 1.0% to 7.5% under optimal temperature and pH conditions, with 1.0% NaCl able to effectively degrade 100 mg/L phenacetin within 80 h. In contrast, at a concentration of 10.0% NaCl, only 16.6% of phenacetin was removed after 80 h (Figure 2D), indicating that despite PNT-23 exhibiting a degree of salt tolerance, high salt concentrations can have a detrimental effect on the efficiency of phenacetin degradation. Overall, understanding the salt tolerance and optimal salt concentrations of PNT-23 is essential for its practical application for phenacetin remediation in diverse contaminated environments.

### 3.3. Identification of Metabolites and Proposed Phenacetin Catabolism Pathway

**Figure 3:**
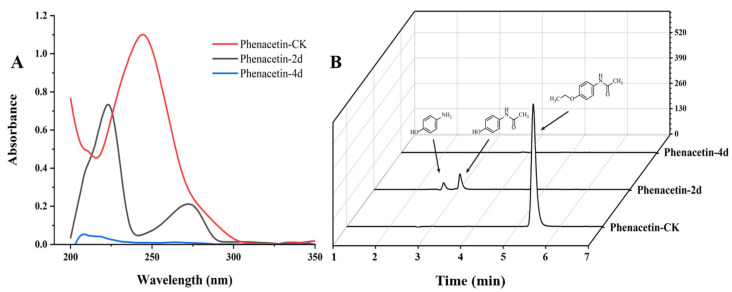
Phenacetin biodegradation profiles: (**A**) change in UV/Vis absorption during phenacetin biodegradation by PNT-23 under optimal degradation conditions; (**B**) HPLC analysis of phenacetin (5.3 min), with two new peaks for N-acetyl-4-aminophenol (3.0 min) and 4-aminophenol (2.7 min).

The degradation of phenacetin by strain PNT-23 was preliminary monitored by UV/Vis spectrophotometry. In Figure 3A, the red line represents the absorption of phenacetin without the addition of strain PNT-23 (phenacetin-CK), while the black and blue lines represent the absorption of samples after 2 days (phenacetin-2d) and 4 days (phenacetin-4d), respectively, with the addition of PNT-23. The maximum wavelength of UV/Vis absorption at 244 nm in phenacetin-CK was considered the maximum absorption peak of phenacetin. During the degradation process, the peak at 244 nm gradually decreased, while new absorption peaks emerged with maximum absorption occurring at 223 nm and 270 nm. Finally, in phenacetin-4d, the new peaks disappeared completely. (Figure 3A).

**Figure 4:**
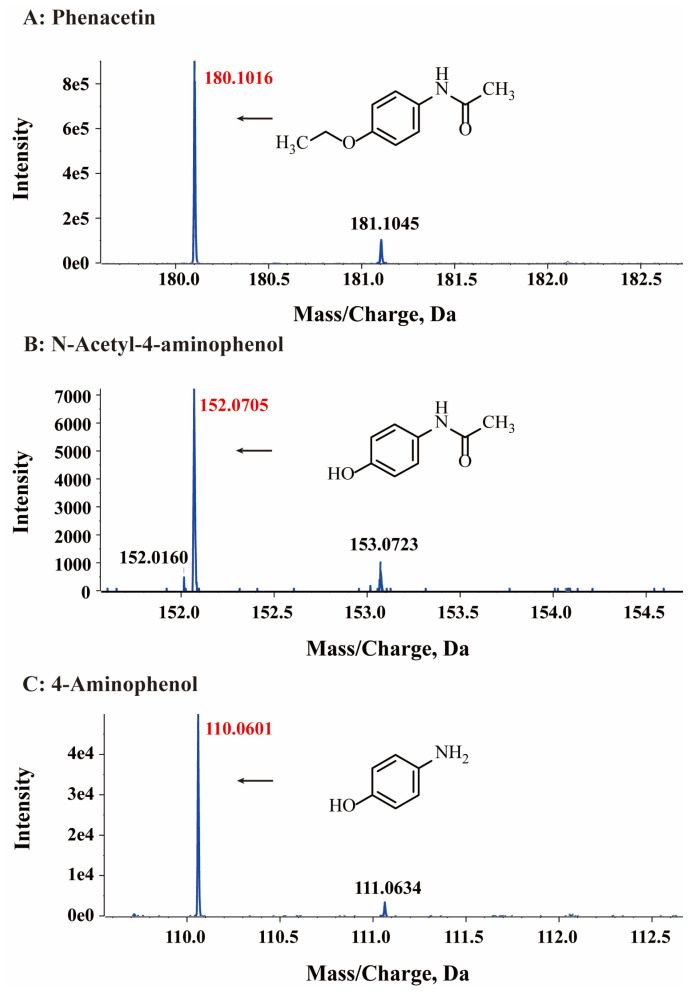
HPLC-MS mass spectra of phenacetin (**A**) and the proposed structures of its intermediates N-acetyl-4-aminophenol (**B**) and 4-aminophenol (**C**).

**Figure 5:**
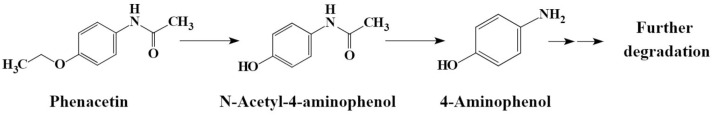
Proposed degradation pathway of phenacetin to 4-aminophenol by *Rhodococcus* sp. strain PNT-23.

HPLC analysis was used to detect the possible catabolic intermediates formed during phenacetin degradation. The phenacetin standard peak had an approximate retention time of 5.3 min. Interestingly, two new peaks emerged at around 4.0 min and 3.6 min, respectively, during the phenacetin degradation processes but finally disappeared (Figure 3B). The elimination of UV/Vis and HPLC absorption peaks indicated the decomposition of phenacetin by strain PNT-23. LC/MS analysis was also performed, with results indicating that the molecular ion peak of phenacetin occurred at m/z 180.1016, in good agreement with the protonated derivative (M+H, C_10_H_14_N_2_O^+^, 180.1020) with error of −2.22 ppm. In addition, two new peaks were observed at 3.0 min and 2.7 min, exhibiting molecular ion peaks at m/z 152.0705 [M+H] and 110.0601 [M+H], respectively. These peaks were found to correspond to the protonated derivatives of N-acetyl-4-aminophenol (M+H, C_8_H_10_N_2_O^+^, 152.0707) and 4-aminophenol (M+H, C_6_H_8_NO^+^, 111.0601) with errors of −1.32 and 0 ppm, respectively (Figure 4). Furthermore, the results of HPLC analysis further confirmed that the two newly formed peaks had retention times corresponding to those of the commercial standards of N-acetyl-4-aminophenol and 4-aminophenol, respectively. Based on these findings, it can be inferred that the two primary metabolites of phenacetin are N-acetyl-4-aminophenol and 4-aminophenol (Figure 5), which are formed from ether bond cleavage and amide bond hydrolysis, respectively [4,40].

Previous studies have shown that, in rodents, guinea pigs and rabbits, phenacetin can be metabolized through de-ethylation, N-deacetylation and ring hydroxylation pathways [41,42,43]. However, in the present study, a distinct metabolic pathway was observed in which *Rhodococcus* sp. PNT-23 converted phenacetin into N-acetyl-4-aminophenol and 4-aminophenol. Interestingly, both intermediates were further metabolized during the experiment, resulting in almost 100% loss of these intermediate compounds. These findings suggest that *Rhodococcus* sp. PNT-23 is capable of efficiently degrading phenacetin and its metabolites. It is worth noting that different microbial strains may utilize varying metabolic pathways for phenacetin degradation. For instance, *Penicillium* species were found to degrade phenacetin into 4-ethoxyaniline and acetate [24], suggesting that different microbial strains utilize varying metabolic pathways.

The fate of 4-aminophenol in *Rhodococcus* sp. PNT-23 is unclear. Microbial conversion of N-acetyl-4-aminophenol has been proposed to proceed via 4-aminophenol to hydroquinone, a major route for biodegradation [25]. In *Pseudomonas moorei* KB4 [44], N-acetyl-4-aminophenol was found to be converted into 4-aminophenol by acyl amidohydrolase, followed by amino group replacement with a hydroxyl group to form hydroquinone [45,46,47], which can be further degraded through various routes [46]. However, in the case of *Rhodococcus* sp. PNT-23, hydroquinone (or other intermediates that may indicate 4-aminophenol hydroxylation to hydroquinone) was not detected. Since no color changes associated with hydroquinone accumulation were observed, and no hydroquinone or other intermediates were detected, 4-aminophenol was likely transformed by PNT-23 through an alternative pathway. In conclusion, while the initial conversion of phenacetin into N-acetyl-4-aminophenol and 4-aminophenol by PNT-23 was clearly observed, the subsequent fate of 4-aminophenol requires further research to confirm the specific pathway in this strain. Confirming the proposed remediation pathway would require comprehensive intermediate identification and isotopic labeling to track the fates of intermediates and end products.

*Rhodococcus* are common bacteria that inhabit a variety of environments, including soil, groundwater, marine sediments, animal feces and plants [48]. The findings of this study not only expand the known substrate spectra of *Rhodococcus* strains but also provide a new candidate for the microbial removal of phenacetin from diverse environments, highlighting the potential value of this genus for bioremediation strategies. Furthermore, the results of this study suggest that optimizing degradation conditions, such as pH, temperature and salt concentrations, can significantly improve the efficiency of phenacetin degradation while reducing the risk of secondary environmental damage. Future studies should focus on identifying strain PNT-23 at the species level and optimizing operational conditions to maximize its ability to degrade phenacetin. Additionally, investigating the detailed mechanisms underlying the phenacetin degradation process could provide a better understanding of the biodegradation pathway, resulting in the development of more efficient bioremediation strategies. These findings have important implications for bioremediation and environmental protection, as well as for the development of safer pharmaceuticals.

## 4. Conclusions

In this study, a novel phenacetin-degrading strain, *Rhodococcus* sp. PNT-23, was successfully isolated from municipal wastewater and maintained in pure culture. The optimal growth conditions for this strain were determined to be a temperature of 37 °C, a pH of 7.0 and a 1% salt concentration. Furthermore, the optimal conditions for phenacetin degradation were found to be a temperature of 30 °C, a pH of 7.0 and a 1% salt concentration. Under these conditions, PNT-23 completely depleted 100 mg/L phenacetin within 80 h. A pathway was proposed for the degradation of phenacetin via N-acetyl-4-aminophenol and 4-aminophenol. However, the metabolic fate of 4-aminophenol and the specific enzymes involved in the degradation process remains unclear and requires further investigation. This study contributes to the current understanding of the microbial diversity and mechanisms of phenacetin biodegradation, contributing to the development of effective and eco-friendly methods for phenacetin removal from the environment.

## Data Availability

Not applicable.

## Supplementary Materials

The following supporting information can be downloaded at: https://www.mdpi.com/article/10.3390/microorganisms11081962/s1, Figure S1: The effect of temperature, pH value and NaCl concentration on the cell growth status of strain PNT-23 in LB medium, (A) the effect of temperature on the cell growth status of strain PNT-23 in LB medium at 16 °C, 20 °C, 25 °C, 30 °C, 37 °C and 44 °C, (B) the effect of pH value on the cell growth status of strain PNT-23 in LB medium at pH values of 5.0, 6.0, 7.0, 8.0, 9.0 and 10.0, and (C) the effect of NaCl concentration on the cell growth status of strain PNT-23 in LB medium at NaCl concentrations ranging from 1% to 10% (*w*/*v*).
