# Supplementary material for: Isolation and Characterization of the Wastewater Micropollutant Phenacetin-Degrading Bacterium Rhodococcus sp. Strain PNT-23
Source: Microorganisms. 2023 Jul 31;11(8):1962. doi: 10.3390/microorganisms11081962 (PMC10458748; doi:10.3390/microorganisms11081962)
Supplement: Supplementary file 1 [file microorganisms-11-01962-s001.zip › microorganisms-2378216-supplementary.pdf]

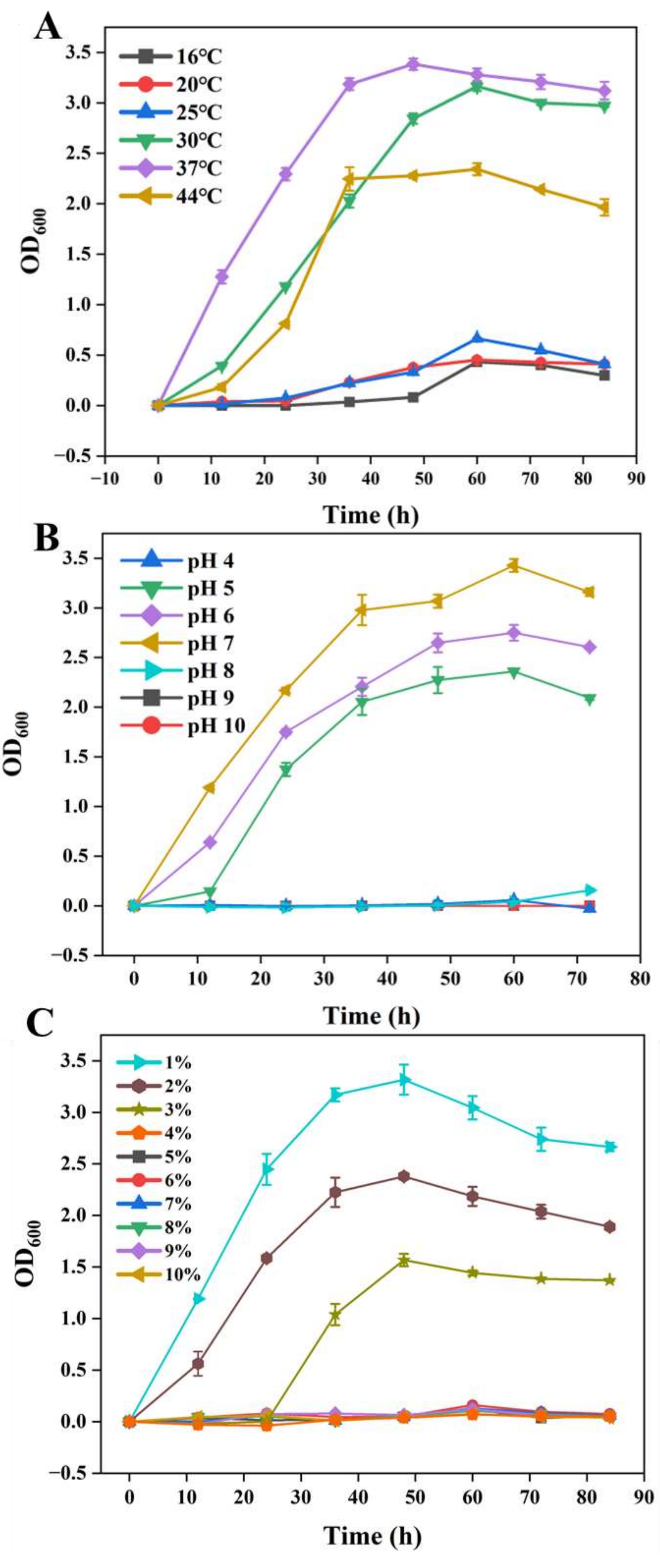

A
16°C
20°C
25°C
30°C
37°C
44°C
OD600
Time (h)
B
pH 4
pH 5
pH 6
pH 7
pH 8
pH 9
pH 10
OD600
Time (h)
C
1%
2%
3%
4%
5%
6%
7%
8%
9%
10%
OD600
Time (h)

Fig. S1: The effect of temperature, pH value and NaCl concentration on the cell growth status of strain PNT-23 in LB medium, (A) the effect of temperature on the cell growth status of strain PNT-23 in LB medium at 16°C, 20°C, 25°C, 30°C, 37°C and 44°C, (B) the effect of pH value on the cell growth status of strain PNT-23 in LB medium at pH values of 5.0, 6.0, 7.0, 8.0, 9.0 and 10.0, and (C) the effect of NaCl concentration on the cell growth status of strain PNT-23 in LB medium at NaCl concentrations ranging from 1% to 10% (*w/v*).
